# The effect of anesthesia without opioid on perioperative opioid demand in children with severe obstructive sleep apnea (OSA) for adenotonsillectomies — single-center retrospective observational study

**DOI:** 10.1186/s40981-022-00530-7

**Published:** 2022-06-14

**Authors:** Connie Mun-Price, Kathleen Than, Margaret J. Klein, Patrick Ross, Eugene Kim, Christian Hochstim, Makoto Nagoshi

**Affiliations:** 1 Department of Anesthesiology and Critical Care Medicine, Children’s Hospital Los Angeles, Keck School of Medicine, University of Southern California, 4650 Sunset Boulevard, Los Angeles, CA 90027 USA; 2 Department of Otolaryngology, Children’s Hospital Los Angeles, Keck School of Medicine, University of Southern California, Los Angeles, CA USA

**Keywords:** Opioid avoidance, Severe OSA, Tonsillectomy, Pediatric

## Abstract

**Background:**

Children with severe obstructive sleep apnea (OSA) carry a higher risk of respiratory complications after adenotonsillectomy. Their altered sensitivity to opioids may be a significant contributor to respiratory morbidity. The purpose of this study was to identify how anesthesia without opioids affects perioperative opioid demand and postoperative course.

**Methods:**

A chart review of children with severe OSA (apnea hypoxia index; AHI ≥ 10) undergoing adenotonsillectomies was performed. Comorbidities and perioperative medications were documented. Perioperative opioid doses within 48 h of procedure were calculated as morphine equivalents (mcg/kg). Pain scores, rescue medications, and postoperative complications in PICU and non-PICU settings were also documented. Anesthesia without opioid and with opioid groups were compared.

**Results:**

The analysis included 225 children. A significantly higher percentage of children received no postoperative opioids in the anesthesia without opioid group compared to those with opioid (46 of 88 children vs. 43 of 137; *P* < 0.05). The incidence of severe postoperative pain between the two groups was not different in PICU (*P* = 0.88) or non-PICU setting (*P* = 0.84). Perioperative opioid administration was significantly lower in anesthesia without opioid (median, Q1, Q3: 0.0, 0.0, 83.0) compared to with opioid (144.4, 72.5, 222.2; *P* < 0.01). Anesthesia without opioid was one of the independent factors to achieve perioperative opioid avoidance (<50mcg/kg).

**Conclusions:**

Anesthesia without opioid for children with severe OSA for tonsillectomy significantly reduced perioperative demand for opioid and did not affect the occurrence of severe pain. Anesthesia without opioid is an effective strategy to minimalize opioid demand perioperatively for children with severe OSA for tonsillectomy.

## Introduction

Adenotonsillectomies are one of the most common surgical procedures performed in children. There have been 3 key issues associated with the management of children with obstructive sleep apnea (OSA) for adenotonsillectomy: preoperative screening, perioperative pain management, and postoperative disposition [1]. Those children with OSA undergoing adenotonsillectomy are at higher risk for respiratory complications postoperatively, especially those with severe OSA [2]. Their altered sensitivity to opioids may be a significant contributor to respiratory morbidity [3]. To date, there have been multiple studies attempting to identify opioid titrating or sparing for children with OSA undergoing adenotonsillectomies using different types of analgesic strategies including reduced opioids, nonsteroidal anti-inflammatory drugs, acetaminophen, ketamine, and dexmedetomidine [4–13]. To address the issue of opioid avoidance, we focused on children with severe OSA, which we believe to be the most vulnerable population to have respiratory complications after adenotonsillectomy. As there is no standardized anesthetic protocol for children with severe OSA undergoing adenotonsillectomy, decisions regarding the use of opioid medications are at the discretion of the anesthesia team. Individual patient characteristics and information available from the preoperative assessment are used to guide this decision-making. The purpose of this study was to identify how anesthesia without opioid affects postoperative opioid demand as a primary outcome and postoperative course including pain, rescue medications, and complications as a secondary outcome in children with severe OSA undergoing adenotonsillectomies.

## Materials and methods

### Data collection

This was an IRB-approved study at Children’s Hospital Los Angeles. Children who underwent adenotonsillectomies were identified through our anesthesia electronic medical record from January 1, 2009, to December 31, 2018. Our study period was divided into prior to January 1, 2014 (2009–2013), and after January 1, 2014 (2014–2018), based upon FDA black box warning regarding codeine, increased literature regarding opioid avoidance at this time, as well as opening of a larger PICU providing more availability in our hospital.

Inclusion criteria were children (younger than 18 years old of age) who underwent adenotonsillectomies and carried a diagnosis of severe OSA defined by a polysomnogram (PSG) with apnea-hypopnea index (AHI) ≥ 10. All the children with severe OSA who underwent adenoidectomy had tonsillectomy. We extracted postoperative medication use as this level of AHI was a trigger for inpatient admission. Children with a tracheostomy were excluded. There were 28 children who were intubated and mechanically ventilated with fentanyl infusion for sedation in PICU. Those were also excluded from our current study due to fentanyl infusion.

Body mass index (BMI) percentile was calculated using the CDC pediatric BMI calculator using sex, age (years), height (meters), and weight (kilograms) at the time of the procedure. Based on BMI percentile, children were classified into 4 categories: BMI % < 5, underweight; 5 ≤ BMI % < 95, normal to overweight; 95 ≤ BMI % ≤ 98, obese; and BMI % ≥ 99, morbidly obese. We chose these BMI ranges separate from the CDC definition as we anticipated a greater degree of obesity may be associated with the decision to avoid opioid medications. Based on AHI from the polysomnogram, children were classified into 3 categories: AHI 10–19.9, AHI 20–39.9, and AHI > 40.

Comorbidities were grouped using the following categories: craniofacial anomaly (cleft palate, craniosynostosis, Treacher-Collins syndrome, Goldenhar syndrome, mucopolysaccharidosis, Apert syndrome, Pfeiffer syndrome, etc.), neuromuscular disease (cerebral palsy, Duchenne muscular dystrophy, mitochondrial disorder, seizure disorder, hydrocephalus, hypotonia, etc.), genetic syndrome (Down syndrome, Apert syndrome, Pfeiffer syndrome, mitochondrial disorders, etc.), congenital heart disease (CHD), and abnormal echocardiogram (ECHO; defined as RV dilation and/or pulmonary HTN). Overlapping categories such as the presence of a syndrome and a craniofacial anomaly were counted in both categories. High-risk factors were picked based on previous reports including morbid obesity (BMI over 99 percentile for age), race = black, AHI > 20, PSG SO_2_ < 80%, PSG EtCO2 > 60 in addition to preexisting craniofacial anomaly, neuromuscular disease, or genetic syndrome [14–17]. Surgical techniques for tonsillectomy were classified by anatomical approach (extracapsular or intracapsular) and device (coblator or non-coblator) [18, 19].

Intraoperative clinical care was provided at the discretion of the anesthesiologist as patients were not under a study protocol. In our hospital, all the children having adenotonsillectomies underwent general anesthesia with an endotracheal tube with sevoflurane without or with opioid administration. Induction of anesthesia was either inhalational, intravenous, or a combination of both. Dexamethasone was routinely administered. Ondansetron was only avoided if requested by the otolaryngologist. Muscle relaxants which required reversal, dexmedetomidine, and/or ketamine administration were recorded.

Our Otolaryngology and Anesthesiology Departments are both involved in the planning for PICU admission. In retrospect, we admitted children to the PICU after tonsillectomy based on factors including higher AHI number (>30), PSG O_2_ Sat. <80%, comorbidities such as difficult airway, obesity, craniofacial anomaly, neuromuscular disease, genetic disorder, severe asthma, chronic lung disease, or congenital heart disease including pulmonary hypertension. These factors were reviewed preoperatively and the PICU bed was reserved typically 1 to 2 days prior to the procedure.

Postoperative destination was recorded as pediatric intensive care (PICU) admission or non-PICU admission (postanesthesia care unit; PACU, then surgical floor admission). None of the children meeting inclusion criteria underwent ambulatory surgery.

Pain assessments for verbal and appropriate children using a Verbal Rating Scale (VRS) or FACES pain scale (0–10) and/or the FLACC pain scale for younger or non-verbal children. For FLACC and VRS, a score of 7–10 was considered severe pain and FACES pain scale of 8–10. In our institution, postoperative administration of opioids was the primary choice to manage children who suffered severe pain. For mild to moderate pain, acetaminophen or nonsteroidal anti-inflammatory drugs (NSAIDs), either ibuprofen or ketorolac, were used in postoperative periods. Opioid administration for mild to moderate pain was considered only after consulting physicians (anesthesiologist, intensivist, or surgeon). The use of ketorolac in our hospital was very limited due to the otolaryngologists’ preference and only administered after consulting and agreement with them postoperatively.

Postoperative opioid administration as needed for pain control was recorded as a rescue opioid. Postoperative complications such as nausea/vomiting, hypoxia requiring airway intervention, prolonged PICU stay, prolonged PACU stay, and prolonged hospital stay were documented. Nausea/vomiting requiring antiemetic agents were counted. Hypoxia requiring airway intervention was defined as SO_2_ lower than 90% requiring airway intervention including the new application of CPAP, increased CPAP setting, increase of supplemental O_2_ flow using facemask/nasal cannula, or use of oral/nasal airway. Prolonged PICU stay was defined as PICU stay over 48 h. Prolonged PACU stay was defined as PACU stay over 3 h with medical reasons such as prolonged demands for high flow supplemental oxygen due to frequent apnea/hypoxia causing SO_2_ lower than 90%. Prolonged hospital stay was defined as hospital stay more than 2 days.

Intraoperative and postoperative opioid administrations within 48 h of the procedure were recorded. Acetaminophen was used both intraoperatively and postoperatively. Nonsteroidal anti-inflammatory drugs (NSAIDs), either ibuprofen or ketorolac, were used in only postoperative periods. The amounts of intraoperative and postoperative opioids (fentanyl, morphine, hydromorphone, oxycodone, hydrocodone, codeine) were calculated and expressed as morphine equivalents per kilogram (mcg/kg) based on the formula: morphine (mg, IV):fentanyl (mg, IV):hydromorphone (mg, IV):hydrocodone (mg, PO):oxycodone (mg, PO):codeine (mg, PO) = 10:0.1:1.5:30:20:200 [20]. Codeine use was completely eliminated after 2013 due to FDA black box warning.

### Statistical analysis

Total perioperative (intraoperative and postoperative) opioid doses administered were compared between anesthesia without opioid and with opioid group using the Mann-Whitney *U* test. Occurrence of postoperative no opioid was compared between anesthesia without opioid and with opioid group using the chi-square test. We examined the occurrence of severe pain and rescue opioid use in PICU and non-PICU settings, then compared between anesthesia without opioid and with opioid group using the chi-square test. Occurrence of postoperative complications was also compared between anesthesia without opioid and with opioid group in PICU and non-PICU settings using the chi-square test.

Independent association with the perioperative opioid avoidance (< 50 mcg/kg) of each perioperative factor was analyzed with chi-square or Fisher’s exact test for categorical variables and the Mann-Whitney *U* test for continuous variables. Variables with independent association (*P* value < 0.10) were considered in the multivariable model selection process.

Backwards stepwise selection was used to determine the final factors associated with postoperative and perioperative opioid sparing; variables were selected if *P* <0.05. Results were expressed as odds ratio, 95% Ward confidence limits (95%CL). All statistical analysis was performed using SAS Version 9.4 for Windows (SAS Institute Inc, Cary, North Carolina).

## Result

**Fig. 1:**
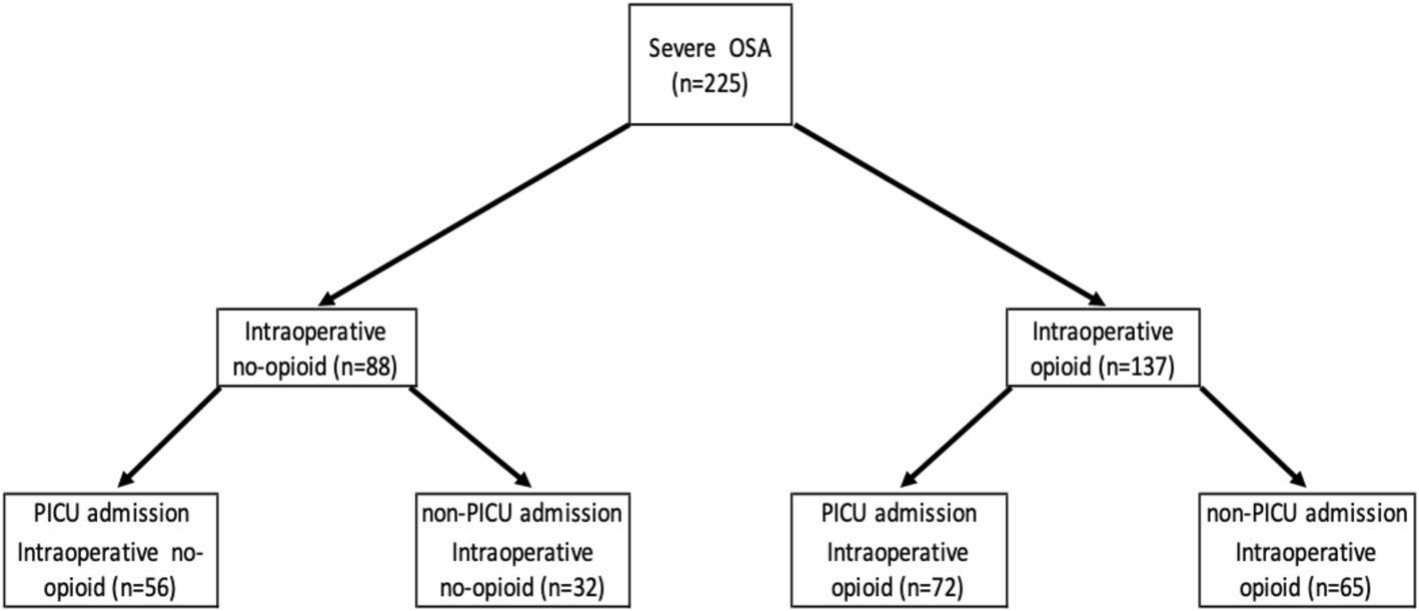
Consort diagram. Two hundred twenty-five children with severe OSA (AHI ≥ 10) were identified. Eighty-eight children underwent anesthesia without opioid and 137 children underwent anesthesia with opioid. Among the anesthesia without opioid group, 56 children were admitted to PICU and 32 children were non-PICU admission. Among the anesthesia with opioid group, 72 children were admitted to PICU and 65 children were non-PICU admission

We identified 225 children with severe OSA (AHI > 10) admitted postoperatively after adenotonsillectomy (Fig. 1). There were 88 children who received anesthesia without opioid and 137 children who received anesthesia with opioid. Among the anesthesia without opioid group, 56 children were admitted to PICU and 32 children were non-PICU admission. Among the anesthesia with opioid group, 72 children were admitted to PICU and 65 children were non-PICU admission. Table 1 shows the demographics of anesthesia without and with opioid group. With respect to surgical technique, all the intracapsular tonsillectomies were carried out using coblation. Comparison of anesthesia groups without opioid and with opioid is shown in Tables 2 and 3. In the comparison of period 2003–2013 (prior to 2014) and period 2014–2018 (after 2013), ketamine use was decreased in the latter (37.3% vs. 26.5%) but dexmedetomidine use increased (13.6% vs. 74.1%). Intraoperative opioid doses (median; Q1, Q3; (morphine equivalent mcg/kg)) of the anesthesia with opioid group were (116.5; 65.3, 182.8) in period 2009–2013, (68.4; 40.3, 119.8) in 2014–2018, and (80.1; 47.4, 129.2) for the entire duration of the study in 2009–2018, respectively. Postoperative opioid doses (median; Q1, Q3; (morphine equivalent mcg/kg)) of the anesthesia without opioid group were (76.3; 6.2, 219.6) in 2009–2013, (0.0; 0.0, 74.1) in 2013–2018, and (0.0; 0.0, 82.3) in 2009–2018 whereas those of the anesthesia with opioid group were (55.4; 17.6, 117.6) in 2009–2013, (34.2; 0.0, 97.5) in 2014–2018, and (39.1; 0.0, 97.6) for the entire duration of the study in 2009–2018. There was no significant difference in postoperative opioid doses between the anesthesia without opioid group and with opioid group in any of the time periods. However, there was a significantly higher percentage of children who did not receive any postoperative opioids in the anesthesia without opioid group compared to the anesthesia with opioid group (46 out of 88 children vs. 43 out of 137; *P* < 0.01). Perioperative opioid doses (median; Q1, Q3) of anesthesia without opioid group were significantly lower than those of the anesthesia with opioid group (76.3; 6.2, 219.6) vs. (177.6; 116.7, 271.8) in 2009–2013 (*P* < 0.05), (0.0; 0.0, 74.1) vs. (120.7; 55.8, 202.8) in 2014–2018 (*P* < 0.01), and (0.0; 0.0, 83.0) vs. (144.4; 72.5, 222.2) in 2009–2018 (*P* < 0.01).

**Table 1:** Demographics of the population studied

	Anesthesia		All
	Without opioid	With opioid	
	*n* = 88	*n* = 137	*n* = 225
**Female;** ***n*** **(%)**	21 (23.9%)	46 (33.6%)	67 (29.8%)
**Age, years;** median (Q1, Q3)	6.9 (4.5, 11.3)	6.6 (4.2, 10.7)	6.8 (4.3, 11.0)
**Weight, kg;** median (Q1, Q3)	35.2 (17.4, 62.0)	28.9 (17.0, 4.7)	31.0 (17.0, 56.4)
**Height, cm;** median (Q1, Q3)	124.0 (100.0, 148.0)	118.5 (100.0, 144.0)	121.0 (100.0, 146.0)
**Race;** ***n*** **(%)**			
Asian/Pacific	1 (1.1%)	3 (2.2%)	4 (4.8%)
Other/unknown	19 (21.6%)	20 (14.6%)	39 (17.3%)
Black	8 (9.1%)	13 (9.5%)	21 (9.3%)
Hispanic	57 (64.8%)	92 (67.2%)	149 (66.2%)
White	3 (3.4%)	9 (6.6%)	12 (5.3%)
**AHI category;** ***n*** **(%)**			
10–20	26 (29.6%)	48 (35.6%)	74 (33.2%)
20–40	31 (35.2%)	54 (40.0%)	85 (38.1%)
≥40	31 (35.2%)	33 (24.4%)	64 (28.7%)
**BMI;** ***n*** **(%)**			
Underweight (<5%)	2 (2.3%)	6 (4.4%)	8 (3.6%)
Normal to overweight (5–95%)	34 (39.1%)	60 (44.1%)	94 (42.2%)
Obese (96–98%)	13 (14.9%)	18 (13.2%)	31 (13.9%)
**Morbidly obese (≥99%)**	38 (43.7%)	52 (38.2%)	90 (40.4%)
**Race: Black;** ***n*** **(%)**	8 (9.1%)	13 (9.5%)	21 (9.3%)
**AHI ≥ 20;** ***n*** **(%)**	62 (70.4%)	87 (64.4%)	149 (66.8%)
**PSG SO2 < 80%;** ***n*** **(%)**	47 (58.8%)	58 (48.7%)	105 (52.8%)
**PSG EtCo2 ≥ 60 mmHg;** ***n*** **(%)**	5 (9.4%)	14 (16.5%)	19 (13.8%)
**Craniofacial anomaly;** ***n*** **(%)**	5 (5.7%)	11 (8.3%)	16 (7.1%)
**Neuromuscular disease;** ***n*** **(%)**	8 (9.1%)	21 (15.3%)	29 (12.9%)
**Genetic syndrome;** ***n*** **(%)**	28 (31.8%)	28 (20.4%)	56 (24.9%)
**Surgical technique;** ***n*** **(%)**			
Extracapsular without coblation	68 (77.3%)	107 (78.1%)	175 (77.8%)
Extracapsular with coblation	4 (4.5%)	13 (9.5%)	17 (7.6%)
Intracapsular with coblation	16 (18.2%)	17 (12.4%)	33 (14.7%)
**PICU admission;** ***n*** **(%)**	56 (63.6%)	72 (52.6%)	128 (56.9%)

“*n*” presented for categorical data unless otherwise stated (median with interquartile range reported for non-normally distributed continuous data). All column percentages are out of the total non-missing. Q1 = 25^Th^ percentile, Q3 = 75^th^ percentile

**Table 2:** Comparison of intraoperative opioid group and no intraoperative opioid group in PICU admission and non-PICU admission

	**Anesthesia**		**All**
	**Without opioid**	**With opioid**	
**2009–2013;** ***n*** **(%)**	*n*=16	*n*=43	*n*=59
Muscle relaxant (rocuronium, vecuronium or cisatracurium)	4 (25%)	22 (51.2%)	26 (44.1%)
Dexmedetomidine only	1 (0.63%)	3 (7.0%)	4 (6.8%)
Both dexmedetomidine/ketamine	4 (25%)	0 (0%)	4 (6.8%)
Ketamine only	9 (28.8%)	9 (20.9%)	18 (30.5%)
NSAIDs	4 (25%)	7 (16.3%)	11 (18.6%)
Acetaminophen	13 (81.3%)	38 (88.4%)	51 (86.4%)
PICU admission	12 (75%)	35 (81.4%)	47 (79.7%)
**2014–2018;** ***n*** **(%)**	*n* = 72	*n* = 94	*n* = 166
Muscle relaxant (rocuronium, vecuronium or cisatracurium)	28 (38.9%)	35 (37.2%)	63 (39.0%)
Dexmedetomidine only	38 (52.8%)	47 (50%)	85 (51.2%)
Both dexmedetomidine/ketamine	28 (38.9%)	10 (10.6%)	38 (22.9%)
Ketamine only	0 (0%)	6 (6.4%)	6 (3.6%)
NSAIDs	32 (44.4%)	27 (28.7%)	59 (35.5%)
Acetaminophen	71 (98.6%)	94 (100%)	165 (99.4%)
PICU admission	44 (61.1%)	37 (39.4%)	81 (48.8%)
**Opioid use (mcg/kg)**	**Anesthesia**		***P*** **value**
Median (Q1, Q3)	**Without opioid (*n*** **= 88)**	**With opioid (*n*** **= 137)**	
Intraoperative			
(2009–2013)	NA	116.5 (65.3, 182.8)	
(2014–2018)	NA	68.4 (40.3, 119.8)	NA
(2009–2018)	NA	80.1 (47.4, 129.2)	
Postoperative			
(2009–2013)	76.3 (6.2, 219.6)	55.4 (17.6, 117.6)	0.96
(2014–2018)	0.0 (0.0, 74.1)	34.2 (0.0, 97.5)	0.09
(2009–2018)	0.0 (0.0, 82.3)	39.1 (0.0, 97.6)	0.06
Perioperative			
(2009–2013)	76.3 (6.2, 219.6)	177.6 (116.7, 271.8)	<0.05^a^
(2014–2018)	0.0 (0.0, 74.1)	120.7 (55.8, 202.8)	<0.001^a^
(2009–2018)	0.0 (0.0, 83.0)	144.4 (72.5, 222.2)	<0.001
**Postoperative no opioid;** ***n*** **(%)**	46 (52.3%)	43 (31.4%)	<0.05

Period 2009–2013 starts from January 1, 2009, to December 31, 2013. Period 2014–2018 starts from January 1, 2014, to December 31, 2018. Total postoperative opioid doses administered were compared between the anesthesia without opioid and with opioid group using the Mann-Whitney *U* test. Occurrence of postoperative no opioid consumptions was compared using the chi-square test

^a^After adjusting for multiple comparisons using the Bonferroni adjustment

**Table 3:** Comparison of intraoperative opioid group and no intraoperative opioid group in PICU admission and non-PICU admission

		PICU admission (*n*=128)			Non-PICU admission (*n*=97)		
		Anesthesia without opioid (*n*=56)	Anesthesia with opioid (*n*=72)	*P* value	Anesthesia without opioid (*n*=32)	Anesthesia with opioid (*n*=65)	*P* value
Severe pain	***n*** **(%)**	28 (50%)	35 (49%)	0.88	13 (41%)	25 (39%)	0.84
Postoperative Rescue opioid	***n*** **(%)**	21 (38%)	50 (69%)	<0.01	21 (66%)	45 (69%)	0.72
Postoperative opioid (mcg/kg)	**Median**	0	39.6	< 0.01	53.1	30.9	0.17
	Q1, Q3	(0, 57.8)	(0, 103.3)		(0, 246.1)	(0, 104.2)	
Postoperative hypoxia requiring airway intervention	***n*** **(%)**	27 (48%)	37 (52%)	0.72	5 (16%)	11 (17%)	0.87
PICU stay > 48 hours or PACU stay > 3h	***n*** **(%)**	2 (4%)	5 (7%)	0.42	12 (38%)	18 (28%)	0.32
Hospital stay > 2 days	***n*** **(%)**	10 (18%)	11 (15%)	0.7	1 (3%)	2 (2%)	0.98

Each incident was compared between the anesthesia without opioid and with opioid groups in PICU setting and non-PICU setting separately using the chi-square test

For children admitted to the PICU, severe pain was reported in 48.6% of the anesthesia without opioid group and in 50.0% of the anesthesia with opioid group (*P* = 0.88). In the PICU, children received postoperative rescue opioid significantly less frequently in the anesthesia without opioid group (37.5%) compared with the anesthesia with opioid group (69.4%; *P* < 0.01). Postoperative opioid dose (median, Q1, Q3) for children in the anesthesia without opioid group (0, 0, 57.8) was significantly lower than those of anesthesia with opioid (39.6, 0, 103.3; *P* < 0.01). For the children admitted to the pediatric ward, severe pain was reported in 38.5% of the anesthesia without opioid group and in 40.6% of the anesthesia with opioid group (*P* = 0.84). In the ward, children received postoperative rescue opioids in equal frequency between the two groups, 65.6% of the anesthesia without opioid group and 69.2% of the anesthesia with opioid group (*P* = 0.72). Postoperative opioid (median, Q1, Q3) of the anesthesia without opioid group (53.1, 0, 246.1) was not significantly different from those of the anesthesia with opioid (30.9, 0, 104.2; *P* = 0.12). In a comparison of postoperative complications between anesthesia without opioid and with opioid groups in both the PICU and non-PICU children, there were no significant differences in the incidence of nausea/vomiting, hypoxia requiring airway intervention, PICU stay over 48 h, PACU stay over 3 h, and hospital stay over 2 days. However, incidence of hypoxia was higher in PICU admission compared to non-PICU admission in both anesthesia without opioid group and with opioid group.

Table 4 shows the results of univariable analysis for factors associated with perioperative opioid avoidance such that the patient received less than 50 mcg/kg of morphine equivalent dose. The differences were significant for AHI > 20 (*P* < 0.05), BMI age percentile over 99% (*P* <0.05), administration of ketamine (*P* < 0.1), dexmedetomidine (*P* < 0.01), neither administration of dexmedetomidine nor ketamine (*P* < 0.01), NSAIDs (*P* < 0.01), PICU admission (*P* < 0.05), anesthesia without opioid (*P* < 0.01), and surgery after 2013 (*P* < 0.01). These factors were used as candidate variables in a forward stepwise selection method.

**Table 4:** Univariable analysis for perioperative opioid avoidance (morphine equivalent dose < 50 mcg/kg)

	Perioperative opioid		
	< 50 mcg/kg	**>** 50 mcg/kg	***P*** value
	***n*** = 77	***n*** = 148	
**Female**	22 (29%)	45 (30%)	0.78
**Age < 3years old**	2 (3%)	25 (17%)	**< 0.01**
**Race Black**	5 (7%)	16 (11%)	0.29
**AHI** **>** **20 (*n*=223)**	59 (77%)	92 (62%)	**<0.05**
**BMI for age** **>** **99% (*n*=223)**	38 (50%)	52 (35%)	**< 0.05**
**PSG SO**_**2**_ **< 80 % (*n*=199)**	40 (56%)	65 (51%)	0.45
**PSG EtCO**_**2**_ **>** **60 mmHg (*n*=138)**	7 (15%)	12 (13%)	0.73
**Craniofacial anomaly**	5 (7%)	11 (7%)	0.79
**Neuromuscular disease**	8 (10%)	21 (14%)	0.42
**Genetic syndrome**	56 (73%)	35 (24%)	0.55
**Congenital heart disease**	68 (88%)	15 (10%)	0.72
**Use of ketamine**	29 (38%)	36 (24%)	**< 0.05**
**Use of dexmedetomidine**	60 (78%)	71 (48%)	**<0.01**
**Neither dexmedetomidine nor ketamine**	11 (14%)	60 (41%)	**<0.01**
**Use of either dexmedetomidine or ketamine**	66 (86%)	88 (60%)	**<0.01**
**NSAIDs (ibuprofen or ketorolac)**	36 (47%)	34 (23%)	**< 0.01**
**Acetaminophen**	74 (96%)	142 (96%)	>0.99
**PICU admission**	52 (68%)	76 (51%)	**< 0.05**
**Anesthesia without opioid**	57 (74%)	31 (21%)	**<0.01**
**Surgery after 2013 (2014–2018)**	70 (91%)	96 (65%)	**<0.01**

Independent association with the perioperative opioid avoidance (< 50 mcg/kg) of each perioperative factor was analyzed with chi-square or Fisher’s exact test for categorical variables and the Mann-Whitney *U* test for continuous variables. Variables with a univariable association *P* < 0.10 were used as candidate variables in a forward stepwise selection method

Table 5 shows final multivariable analysis of independent factors for perioperative opioid avoidance (total dose less than 50 mcg/kg); surgery after 2013 (odds ratio, 95% CI, *P* value; 5.26, 1.79–14.29, *P* < 0.01), anesthesia without opioid (11.1, 5.26–20.0, *P* < 0.01), and AHI > 20 (2.60, 1.20–5.62, *P* < 0.05).

**Table 5:** Multivariable logistic regression analysis for perioperative opioid avoidance (total morphine equivalent dose < 50 mcg/kg)

Odds ratio estimates				
Effect	Point estimate	95% Wald confidence limits		***P*** value
**Surgery after 2013**	5.26	1.79	14.29	*P* < 0.01
**Anesthesia without opioid**	11.1	5.26	20.0	*P* < 0.01
**AHI** **>** **20**	2.60	1.20	5.62	*P* < 0.05

The odds of perioperative opioid avoidance are increased with surgery after 2013, anesthesia without opioid, and AHI > 20. The odds are decreased with the early surgery date (prior to 2014) (when controlling for everything else in the model)

## Discussion

Postoperative pain management in severe OSA patients is challenging, especially after airway procedures such as adenotonsillectomies. If treated insufficiently, postoperative pain after adenotonsillectomy may cause emergence agitation in children which may trigger bleeding from operative sites. On the contrary, children may be too sedated with opioids to maintain airway patency and/or to maintain respiratory drive due to central apnea. Some of the reported severe adverse events after adenotonsillectomies may have been associated with increased opioid sensitivity in severe OSA patients as well as overdosing caused by the use of codeine [2, 3, 21, 22]. In the previously published literature, dexmedetomidine [6, 23], ketamine [7], and acetaminophen [24] have been used for children with OSA undergoing adenotonsillectomies and have been shown to have some ability to reduce the use of opioids. There were several factors that likely contributed to a difference in our opioid administration in anesthesia based on epoch of the surgery. The published FDA Blackbox warning of codeine in pediatric use was in 2013. In our facility, there was the introduction of prefilled 3-ml dexmedetomidine syringe in 2013. There were multiple studies published raising awareness of the risk of OSA and opioid use after post-tonsillectomy [2, 3, 25, 26]. Finally, additional PICU beds became available in 2014 at our facility. Therefore, in designing the study, we anticipated a potential shift in pain management before and after 2014 with increased incentive to avoid opioids.

Different surgical techniques may also contribute to decreasing postoperative pain. Use of coblator over non-coblator dissection [19] and intracapsular over extracapsular approach for tonsillectomy [18] may decrease pain postoperatively. In the comparison of the anesthesia without and with opioid groups, the use of surgical techniques was similar. Extracapsular tonsillectomy without coblation was used (77.3% vs. 78.1%). Therefore, difference in surgical techniques seems not to affect the comparison in two groups.

In this study, we found that our pediatric anesthesiologists avoided the use of intraoperative opioids for children with severe obstructive sleep apnea with an AHI ≥ 20, morbidly obesity (BMI > 99 percentile of age), PSG SO_2_ < 80%, genetic syndrome, and children anticipated to be admitted to the PICU postoperatively. For children in which opioids were avoided, alternative medications used to help manage postoperative pain included NSAIDs, dexmedetomidine, and ketamine. Acetaminophen was given equally and frequently to all children.

Our results indicated anesthesia without opioid significantly decreased the perioperative opioid demands. We designed perioperative avoidance as total morphine equivalent dose < 50 mcg/kg based on the recommended maximal of intravenous morphine bolus dose. Perioperative opioid avoidance was significantly higher in the anesthesia without opioid group in PICU setting (*P* < 0.01). Multivariable analyses identified anesthesia without opioid as one of the independent factors for perioperative opioid avoidance, further supporting the role of anesthesia without opioid as a strategy to decrease perioperative opioid demand. AHI > 20 was the only statistically significant. We did not find other perioperative factors that were statistically associated with perioperative opioid avoidance. Patient characteristics including craniofacial anomalies, neuromuscular disease, genetic syndromes, congenital heart disease, and findings from polysomnogram recordings except AHI were not statistically associated with the decision to avoid opioids.

It can be assumed that children at higher risk for perioperative complications are more frequently admitted postoperatively to the PICU. Therefore, there may be a stronger hesitancy by anesthesiologists administering intraoperative opioid to children who will be admitted to PICU postoperatively [2, 3, 25, 26]. In our study, 43.8% of children admitted to PICU underwent anesthesia without opioid. Among the non-PICU children admitted to the ward postoperatively, only 33.0% of children underwent anesthesia with opioid. There was no significant difference in postoperative severe pain scores between children who underwent anesthesia without opioid and anesthesia with opioid regardless of PICU or non-PICU (PACU then ward) admission. In the PICU, rescue opioid was given less frequently in the anesthesia without opioid group compared with the anesthesia with opioid group (*P* < 0.01). For children admitted to the PICU who underwent anesthesia without opioid, the plan to limit opioids was carried through by the team and the use of rescue opioids was limited. However, for children admitted to the regular ward, there was no significant difference in rescue opioid use between the two groups.

As for postoperative complications, opioids, ketamine, dexmedetomidine, or NSAIDs may play a role. Opioids may be responsible for nausea/vomiting, delirium, or delay of emergence. Emergence delirium may be associated with administration of ketamine, while dexmedetomidine may cause delayed emergence. NSAIDs, especially ketorolac, may cause bleeding from the surgical sites. However, neither emergence delirium nor postoperative bleeding was recorded within 48 h of postoperative period. Dexmedetomidine was administered more in the anesthesia without opioid group than in the anesthesia with opioid group. However, the incidences of prolonged PICU/PACU stay or prolonged hospital stay was not different between anesthesia without opioid and with opioid groups. There were no significant differences in incidences of nausea/vomiting, hypoxia requiring airway intervention, prolonged PICU/PACU stay, or prolonged hospital stay between the anesthesia without opioid and with opioid groups either in PICU or in non-PICU setting.

There were several limitations in this study. This was a retrospective observation study over an 8-year period. As a retrospective study, our severe OSA patients for tonsillectomy were managed with different anesthetic techniques based on personal preference of the anesthesia practitioners. It is conceivable that perioperative management varied over the course of the study period. As mentioned above, our study observation period was divided into prior to 2014 and period thereafter (including 2014 to 2018). Although we tried collecting information from medical records as accurately as possible, there could have been some inconsistency in useful information. For example, occasionally, only a FLACC pain scale was recorded in the electronic medical record even though other pain scales (VRS, FACES) were also used to determine the administration of opioids for severe pain. We do not have recorded measures of emergence delirium which may affect the use of postoperative opioid medications. However, as a practice, we typically use other medications to treat emergence delirium. Intensiveness of care differs among PICU, PACU, and regular ward. Therefore, postoperative pain management may be also different among units based on the individual unit’s level of care. This study could only demonstrate anesthesia without opioid as an independent factor for perioperative opioid avoidance but could not demonstrate administration of either ketamine or dexmedetomidine as an independent factor. This can be explained by the high correlation between anesthesia without opioid and use of dexmedetomidine and/or ketamine.

For the sample size and power analysis based on the Wilcoxon-Mann-Whitney test, we found that our current sample size, sample mean, and standard deviations were well powered in the perioperative comparison but underpowered in the postoperative comparison.

As for the chi-square test to compare no-postoperative opioid managements of the anesthesia without opioid and with opioid groups, both sample size and power (88%) for the comparison were sufficient. Finally, this is a single-center study, so the results observed here may not be generalizable to other institutions, and due to the exploratory nature of the analysis, all the results should be interpreted cautiously.

## Conclusion

Our retrospective observation showed that anesthesia without opioid in the management of children with severe OSA (AHI > 10) for tonsillectomy neither increased postoperative opioid demand nor increased occurrence of severe pain. Multiple predictive factors were identified that were associated with perioperative opioid avoidance (morphine equivalent dose < 50 mcg/kg) in children with severe OSA after adenotonsillectomy: surgery after 2013, anesthesia without opioid, and AHI ≥ 20. These factors may identify the anesthesiologist’s concern for perioperative respiratory depression in choosing a management strategy.

## Data Availability

The datasets used and/or analyzed during the current study are available from the corresponding author on reasonable request.
